# Clinical identification of patients readmitted to hospitals: why patients return

**DOI:** 10.1186/1756-0500-6-419

**Published:** 2013-10-18

**Authors:** Ronald J Lagoe, Diane Nanno, Mary Luziani

**Affiliations:** 1 Hospital Executive Council, PO Box 35089, Syracuse, NY 13235, USA; 2 Transition Care Services, Upstate University Hospital, Syracuse, NY 13210, USA; 3 Care Management, St. Joseph’s Hospital Health Center, Syracuse, NY 13203, USA

**Keywords:** Hospital readmissions, Hospital outcomes, Heart failure, Pneumonia

## Abstract

**Background:**

Increased interest in hospital outcomes has supported the need for clear and useful identification of patients who are readmitted. These patients have frequently been identified by the principal diagnosis of the initial admission.

**Findings:**

In order to evaluate the effectiveness of identifying patients who were subsequently readmitted, those with two frequently encountered conditions, principal diagnoses of congestive heart failure and pneumonia, in the hospitals of Syracuse New York were evaluated. Both populations had large proportions of readmissions involved with principal diagnoses other than pneumonia. For patients with heart failure, a majority of readmitted patients had other diagnoses for two of the hospitals. For patients with pneumonia, a majority of patients had other diagnoses for all of the hospitals.

**Conclusions:**

The data suggested that many patients who were subsequently readmitted are best identified as medicine patients with multiple diagnoses, rather than a single one. This approach addresses the need to manage a wide range of conditions for hospital readmissions, rather than a narrow approach on individual diagnosis.

## Background

In the United States and many Western nations, the need for containment of health care costs is rising [1]. Between 2000 and 2010, increases in per capita health care expenses reached 71.9 percent in the United States, 76.4 percent in Canada, 61.9 percent in Germany, and 87.2 percent in the United Kingdom. Expenses were substantially higher in the United States than in the other nations throughout this period [2]. Recent discussions concerning containment of health care expenditures have generated a high level of interest in connecting a portion of provider reimbursement to outcomes indicators such as hospital inpatient readmissions and complications [3].

In this context, hospital readmissions have generated considerable attention. Reduction of readmissions holds the potential for elimination of large numbers of inpatient hospitalizations and the payments associated with them [4,5]. Further payer revenue could be generated through financial penalties for excess readmissions by providers whose rates could not be reduced. The Medicare program has already developed and implemented financial penalties based on excess readmission levels [3,6-8].

This increased interest in hospital outcomes has supported the need for clear and useful clinical definitions and designations of patients who are readmitted. Historically, these patients have frequently been identified by the principal diagnosis of the initial admission that preceded the readmission, such as heart failure, pneumonia, or acute myocardial infarction [9,10]. Such designations are necessary for effective identification and management of these patients. The potential for developing such definitions has also been supported by the development of computerized algorithms to track and evaluate readmissions using provider data [11].

This brief report focused on evaluation of clinical definitions and designations of patients who are readmitted to hospitals. Through data from hospitals in one community, it evaluated examples of two major diagnoses of these patients and suggested implications for their management.

The study involved readmissions in the hospitals of Syracuse, New York. These acute care facilities, with 2012 inpatient discharges excluding well newborns, include Crouse Hospital (20,715), St. Joseph’s Hospital Health Center (25,743), and Upstate University Hospital (27,600). The primary and secondary acute care service area for these hospitals includes a population of approximately 575,000. The combined hospitals also serve as the tertiary referral center for the eleven county Central New York Health Service Area with a population of 1,405,221 (New York Statistical Information System, 2012).

Historically, the Syracuse hospitals have employed a combination of cooperation and competition to improve health care efficiency and outcomes in the service area. A number of cooperative efforts have been developed through the Hospital Executive Council, the collaborative planning organization for the hospitals [12].

This study was developed through a partnership between the Hospital Executive Council and 3 M™ Health Information Systems. It involved analysis of hospital readmissions in the Syracuse hospitals during a two year period using the 3 M™ HIS Potentially Preventable Readmissions software [13].

## Findings

Provider studies of patients readmitted to hospitals has frequently identified these patients by the principal diagnosis of the initial admission that preceded the rehospitalizations. This approach has frequently caused efforts to manage these outcomes to be based on that diagnosis. This designation has also been reflected in Medicare’s readmission payment methodology, the first phase of which involved patients with initial admissions for heart failure, pneumonia, and acute myocardial infarction [14,15].

In order to evaluate the effectiveness of this approach to identifying patients who are subsequently readmitted, two of these populations were evaluated in the Syracuse hospitals. Patients with a principal diagnosis of congestive heart failure (APR DRG 194) and pneumonia (APR DRGs 138–139) are two diagnoses frequently associated with these patients. Both diagnoses were used to identify patients in the Medicare readmissions program.

For patients with each of these APR DRGs on initial admission, data identifying readmission APR DRGs for each of the three Syracuse hospitals were generated for January-December 2011 and 2012. Combined data for the three hospitals were also identified. The analysis focused on comparison of readmission APR DRGs with initial admission APR DRGs for each hospital by year and quarter during the two year period.

The first section of the analysis involved comparisons of initial and readmission diagnoses for patients with an initial admission for heart failure during the two year period. Relevant data are summarized in Table 1.

**Table 1:** Principal diagnosis of readmission, initial admission-congestive heart failure (APR DRG 194), Syracuse hospitals, 2011-2012

	**Number of readmission**						**Percent of total**	
	**Congestive**	**Other**	**Respiratory**	**Kidney/urinary**	**Digestive**	**Total all**	**CHF**	**All other**
	**heart failure**	**cardiology MDC 5**	**system MDC 4**	**tract MDC 11**	**system MDC 6**	**readmission**		
Crouse Hospital								
1Q 2011	3	1	4	1	0	9	33.33	66.67
1Q 2012	18	1	3	0	3	27	66.67	33.33
2Q 2011	7	1	3	2	0	13	53.85	46.15
2Q 2012	3	0	2	2	0	7	42.86	57.14
3Q2011	10	0	1	1	0	13	76.92	23.08
3Q 2012	6	3	1	0	1	14	42.86	57.14
4Q 2011	7	1	2	1	0	15	46.67	53.33
4Q 2012	4	0	2	2	2	10	40.00	60.00
Total 2011	27	3	10	5	0	50	54.00	46.00
Total 2012	31	4	8	4	6	58	53.45	46.55
St Joseph’s Hospital Health Center								
IQ 2011	7	3	3	1	0	13	5385	46.15
1Q 2012	9	2	6	3	1	24	37.50	62.50
2Q 2011	8	5	4	3	2	33	24.24	75.76
2Q 2012	9	4	2	2	2	22	40.91	59.09
3Q 2011	15	2	4	1	1	25	60.00	40.00
3Q 2012	20	4	10	2	0	43	46.51	53.49
4Q 2011	12	2	6	3	1	27	44.44	55.56
4Q 2012	21	4	12	3	5	59	35.59	64.41
Total 2011	42	12	17	8	4	98	4286	57.14
Total 2012	59	14	30	10	8	148	39.86	60.14
Upstate University Hospital-Main Campus								
1Q 2011	2	1	1	0	1	6	33.33	66.67
1Q 2012	2	1	4	1	4	14	14.29	85.74
2Q 2011	3	0	6	0	1	11	27.27	72.73
2Q 2012	4	1	1	0	1	8	50.00	50.00
3Q 2011	6	0	2	0	0	11	54.55	45.45
3Q 2012	1	0	0	0	0	4	25.00	75.00
4Q 2011	7	1	3	1	1	13	53.85	46.15
4Q 2012	3	0	2	1	0	7	42.86	57.14
Total 2011	18	2	12	1	3	41	43.90	56.10
Total 2012	10	2	7	2	5	33	30.30	69.70
Combined Hospitals								
1Q 2011	12	5	8	2	1	28	42.86	57.14
1Q 2012	29	4	13	4	8	65	44.62	55.38
2Q 2011	18	6	13	5	3	57	31.58	68.42
2Q 2012	16	5	5	4	3	37	43.24	56.76
3Q 2011	31	2	7	2	1	49	63.27	36.73
4Q 2011	26	4	11	5	2	55	47.27	52.73
Total 2011	87	17	39	14	7	189	46.03	53.97
Total 2012	100	20	45	16	19	239	41.84	58.16

These data demonstrated that readmissions at each of the hospitals involved a wide range of principal diagnoses. For St. Joseph’s Hospital Health Center and University Hospital, larger proportions of readmissions for heart failure had other principal diagnoses. These percentages were slightly higher in 2012 than 2013, 60 versus 57 percent for St. Joseph’s Hospital and 69 versus 56 percent for University Hospital. For Crouse Hospital, the percentage of readmissions with heart failure comprised a slight majority (53 – 54 percent) for both years. For the combined hospitals, the proportion of readmissions with heart failure was 41.8 percent of the total.

The quarterly comparisons varied somewhat at each of the hospitals. At St. Joseph’s Hospital Health Center, non heart failure readmissions comprised a majority for all four quarters of 2012 and two quarters of 2011. For University Hospital, the pattern was similar. For Crouse Hospital, non heart failure readmissions comprised a majority for three quarters of 2012 and two quarters of 2011. For the combined hospitals, non heart failure readmissions ranged from 36 – 68 percent.

For readmissions with principal diagnoses other than heart failure, the range of diagnoses was also wide. Respiratory diagnoses comprised the largest proportion at each of the hospitals and the combined total, with more readmissions than other cardiology at all three facilities. Kidney and urinary tract diagnoses and digestive disorders also accounted for readmissions.

These data may suggest the distribution of readmissions for patients with a chronic diagnosis that has been used widely to address this outcome. The second component of the study focused on the clinical identification of patient with pneumonia, an infectious disease, in the Syracuse hospitals, who were subsequently readmitted. Relevant data are summarized in Table 2.

**Table 2:** Principal diagnosis of readmission, initial admission - pneumonia (APR DRGs 138-139), Syracuse hospital, 2011-2012

	**Percent of total**							
	**Pneumonia**	**Other respiratory**	**Circulatory system**	**Infectious diseases**	**Digestive system**	**Total all**	**Pneumonia**	**All other**
		**MDC 4**	**MDC 5**	**MDC 18**	**MDC 6**	**readmissions**		
Crouse Hospital								
1Q 2011	2	4	1	0	1	9	22.22	77.78
1Q 2012	0	5	1	0	1	8	0.00	100.00
2Q 2011	0	0	1	0	1	2	0.00	100.00
2Q 2012	0	0	5	1	0	8	0.00	100.00
3Q 2011	0	1	0	0	0	2	0.00	100.00
3Q 2012	0	0	0	1	1	2	0.00	100.00
4Q 2011	0	1	2	1	0	7	0.00	100.00
4Q 2012	1	1	2	0	0	7	14.29	85.71
Total 2011	2	6	4	1	2	20	10.00	90.00
Total 2012	1	6	8	2	2	25	4.00	96.00
St Joseph’s Hospital Health Center								
1Q 2011	5	8	3	2	0	26	19.23	80.77
1Q 2012	3	9	3	2	5	24	12.50	87.50
2Q 2011	5	6	4	2	1	21	23.81	76.19
2Q 2012	5	6	3	0	1	19	26.32	73.68
3Q 2011	3	7	4	4	2	22	13.64	86.36
3Q 2012	4	3	2	2	1	18	22.22	77.78
4Q 2011	3	5	5	0	1	18	16.67	83.33
4Q 2012	4	8	3	0	3	23	17.39	82.61
Total 2011	16	26	16	8	4	87	18.39	81.61
Total 2012	16	26	11	4	10	84	19.05	80.95
Upstate University Hospital-Main Campus								
1Q 2011	1	1	0	0	0	4	25.00	75.00
1Q 2012	4	0	1	0	0	6	66.67	33.33
2Q 2011	1	1	0	0	0	3	33.33	66.67
2Q 2012	2	0	0	1	0	4	50.00	50.00
3Q 2011	0	0	2	0	1	2	0.00	100.00
3Q 2012	0	0	0	1	1	3	0.00	100.00
4Q 2011	1	2	0	0	1	4	25.00	75.00
4Q 2012	0	0	0	0	0	0	0.00	0.00
Total 2011	3	4	2	0	2	13	23.08	76.92
Total 2012	6	0	1	2	1	13	46.15	53.85
Combined Hospitals								
1Q 2011	8	13	4	2	1	39	20.51	79.49
1Q 2012	7	14	5	2	6	38	18.42	81.58
2Q 2011	6	7	5	2	2	26	23.08	76.92
2Q 2012	7	6	8	2	1	31	22.58	77.42
3Q 2011	3	8	6	4	3	26	11.54	88.46
3Q 2012	4	3	2	4	3	23	17.39	82.61
4Q 2011	4	8	7	1	2	29	13.79	86.21
4Q 2012	5	9	5	0	3	30	16.67	83.33
Total 2011	21	36	22	9	8	120	17.50	82.50
Total 2012	23	32	20	8	13	122	18.85	81.15

These data demonstrated that readmissions for patients with pneumonia at each of the hospitals also involved a wide range of principal diagnoses. At each of the hospitals, a majority of readmissions were for patients with diagnoses other than pneumonia. These proportions ranged from 90 – 96 percent at Crouse Hospital, to 80 – 81 percent at St. Joseph’s Hospital Health Center, to 53 – 76 percent at University Hospital to 81 – 82 percent for the combined hospitals.

As in the case of congestive heart failure, the quarterly comparisons varied somewhat from these values, however, almost all of them demonstrated that high proportions of readmissions involved patients with principal diagnoses other than pneumonia. This was most apparent at Crouse Hospital, where 77 – 100 percent of the quarterly readmissions and St. Joseph’s Hospital Health Center, where 73 – 87 percent of quarterly readmissions occurred outside pneumonia. For the combined hospitals 76 – 88 percent of quarterly readmissions occurred outside pneumonia.

The study data indicated that the readmissions which did not involve pneumonia included a considerable range of diagnoses. For Crouse Hospital, St. Joseph’s Hospital Health Center, and the combined hospitals, the largest numbers involved chronic respiratory diagnoses such as chronic obstructive pulmonary disease and chronic circulatory diagnoses such as heart failure and cardiac arrhythmia. Other diagnoses involved infectious diseases such as septicemia and digestive disorders such as gastroenteritis.

The study demonstrated that, although the range of readmission diagnoses for patients with both heart failure and pneumonia in the Syracuse hospitals was considerable, larger percentages of pneumonia readmissions had different principal diagnoses. This finding may have resulted from the fact that heart failure is a chronic disease, where patients typically experience repeat episodes and are at risk for additional hospitalizations, while pneumonia is an infectious disease that does not usually generate repeat episodes.

## Conclusions

This study demonstrated that, for patients with an initial diagnosis of heart failure or pneumonia who were subsequently readmitted in the Syracuse hospitals, readmissions involved a wide range of principal diagnoses. For both heart failure and pneumonia patients, patients who were readmitted had diagnoses other than these conditions.

These data suggested that, if the data from the Syracuse hospitals are typical, it is probably misleading to characterize readmissions in the context of a single diagnosis. The data indicate that the Syracuse patients with heart failure or pneumonia on their initial admission were frequently adult medicine patients with combinations of circulatory, respiratory, renal, and other medical diagnoses. For many of these patients, the principal diagnosis of heart failure or pneumonia may have been assigned because of the need to designate a single principal diagnosis for payment.

Recent initiatives by health care payors that include financial penalties for hospital readmissions are based on the principal diagnosis of the readmission, without regard to the principal diagnosis of the initial admission. It is theoretically possible that hospitals could attempt to avoid these penalties by coding patients in a different diagnosis. If, however, identification of readmissions is based on all adult medicine patients, the penalties would be difficult to avoid. This is another reason for identifying readmissions according to wide clinical services rather than individual diagnoses.

As hospitals in the United States work to reduce inpatient readmissions in order to improve care and avoid financial penalties, these data have important implications for patient management. They suggest that many patients who are subsequently readmitted are best characterized as medicine patients, with multiple diagnoses rather than a single one. This kind of description addresses the need to manage a wide range of conditions, rather than exacerbation of a single initial diagnosis. It will also require a broad approach to patient management, rather than a narrow focus of resources on a single admission diagnosis.

The same logic suggests the need for health care payers such as Medicare to characterize patients at risk of readmission with respect to medicine or other services, rather than individual diagnoses. As payers increase the number of diagnoses addressed by their programs, their target populations will probably utilize resources in the same manner as adult medicine or other clinical services. This will occur as the size of the patient populations addressed approach the size of the patient populations for whole clinical services. The result of these adjustments will mean that the utilization generated by hospital readmissions will more closely approach the real world of patient management and clinical practice.

This study should encourage participants in the health care system to identify hospital readmissions based on large clinical services rather than specific diagnoses. For providers this will result in a more realistic approach to identification of these outcomes. For payors, it will result in a better justification for financial penalties, if appropriate.

## Availability of supporting data

The data used to develop this manuscript were provided by the individual Syracuse hospitals to the Hospital Executive Council Data Repository. Because this information concerns hospital readmissions, it includes unique patient identifiers. The Hospital Executive Council can provide limited access to this information for individual researchers who contact the Council regarding this subject.

## Competing interests

The authors declare that they have no competing interests.

## Authors’ contributions

RL was responsible for the design of the community wide study and development of results. DN and ML were responsible for review of hospital data and development of conclusions. All authors read and approved the final manuscript.
